# The Role of Adjuvant Systemic and Intravitreal Corticosteroids in Fungal Endophthalmitis Treatment

**DOI:** 10.3390/jof9121147

**Published:** 2023-11-28

**Authors:** Jamal Azhari, Pedro S. Tetelbom, Ahmed B. Sallam

**Affiliations:** Department of Ophthalmology, Harvey and Bernice Jones Eye Institute, University of Arkansas for Medical Sciences, Little Rock, AR 72205, USA; joazhari@uams.edu (J.A.); pstetelbom@uams.edu (P.S.T.)

**Keywords:** fungal endophthalmitis, inflammatory response, pathobiology, systemic corticosteroids, intravitreal corticosteroids, *Candida*, molds, yeast, *Aspergillus*, antifungals

## Abstract

Endophthalmitis refers to inflammation involving internal ocular structures, including the anterior and posterior eye segments, associated with infectious agents, most commonly bacteria and fungi. This review focuses on endophthalmitis caused by fungi. Medical and surgical management are the two main treatment modalities for fungal endophthalmitis, with medical management utilizing systemic or intravitreal antifungals. The use of systemic or intravitreal corticosteroids as an adjuvant treatment to dampen the severity of inflammation is controversial. Based on the pathobiology of fungal endophthalmitis as well as the mechanism of action of corticosteroids, it was hypothesized that corticosteroids affected the immune response against fungal infection. In vitro studies mostly carried out during the 1980s showed that dexamethasone plays a role in the suppression of phagocytosis of yeasts and demonstrated the facilitation of yeast proliferation by dexamethasone. In vivo studies analysis was compromised entirely of retrospective studies describing steroid use in fungal endophthalmitis, with the outcomes of the patients in these studies varying greatly and often being anecdotally noted, thus difficult to discern any definitive results. Given the limited clinical data and the heterogeneity of the existing studies, additional experimentation human studies with clinical trials or observations over more extended periods analyzing the effect of systemic and intravitreal corticosteroids in fungal endophthalmitis are needed before definitive conclusions can be drawn.

## 1. Introduction

Endophthalmitis refers to inflammation involving internal ocular structures including the anterior and posterior segments of the eye, associated with infectious agents, most commonly bacteria and fungi [1]. Endophthalmitis, regardless of the cause, can be characterized by liquefaction of the vitreous with inflammatory cellular infiltration, and severe disease can lead to the formation of fibrous membranes, with fungal endophthalmitis also associated with a granulomatous component. Culture can determine the specific etiology of endophthalmitis in only up to 60% of cases; however, certain clinical findings exist, such as vitreous “snowballs” being more indicative of fungal infection, as well as a more indolent course being associated with fungal causes versus the rapid progression of bacterial endophthalmitis [1].

Exogenous endophthalmitis and endogenous endophthalmitis exist as two distinct etiologies for the development of the disease, with different clinical signs and risk factors [1]. Exogenous fungal endophthalmitis, the most common type of fungal endophthalmitis, has been divided into categories based on the inciting cause, including post-intraocular surgery/procedure, keratitis-associated, and post-penetrating trauma [1]. While not well studied, there is no strong association with any underlying systemic condition or medical disease [1]. Multiple different fungi have been implicated in the disease process, with the estimated incidences varying widely based on the report. Wykoff and colleagues conducted research on 41 instances of culture-positive exogenous fungal endophthalmitis [2]. Their findings revealed that molds, primarily *Aspergillus* and fusarium species, were responsible for 35 cases (constituting 85% of the cases), while yeasts, specifically *Candida* species, accounted for the remaining 6 cases (approximately 15%) [2]. In a separate non-comparative retrospective case series conducted by Silva et al., which focused on culture-confirmed exogenous cases, the most prevalent organisms involved were *Candida*, fusarium, and *Aspergillus* [3]. Their study examined a total of 47 isolates from patients with exogenous fungal endophthalmitis, revealing that 14 (29.8%) of these cases were attributed to *Candida* species, 10 (21.3%) to fusarium species, and 8 (17.0%) to *Aspergillus* species [3].

**Figure 1:**
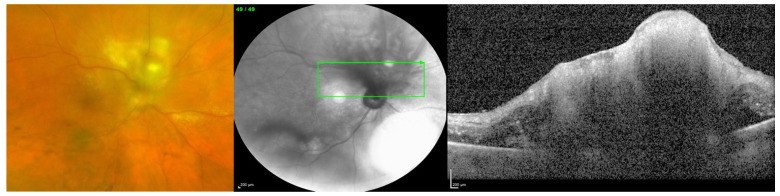
Endogenous fungal endophthalmitis in a patient with a history of IV substance use. Color fundus photo (**left**) showing vitreous haze and a retinal lesion with surrounding retinal edema and exudates. Near-infrared image (**middle**) and optical coherence tomography (**right**) of the same eye showing an irregular lesion of the retina and the underlying choroid.

**Figure 2:**
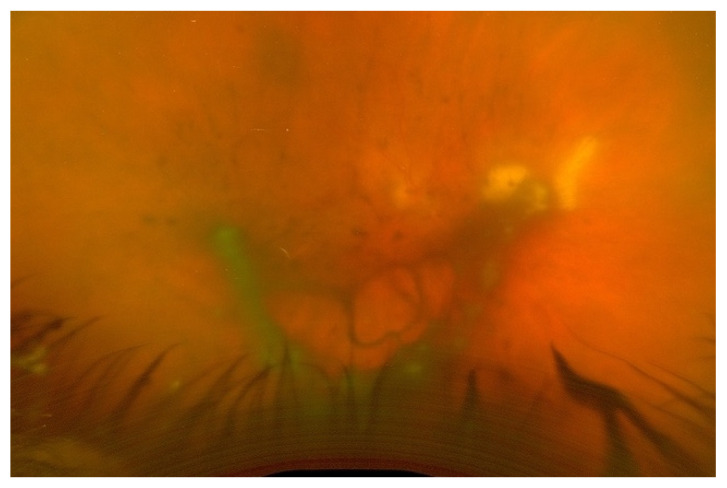
Endogenous fungal endophthalmitis in a patient with multiple bowel surgeries and progressive bilateral vision loss. Wide-field fundus photo of the right eye shows characteristic creamy-white lesions in the retina and vitreous opacities.

Endogenous endophthalmitis refers to the hematogenous dissemination of organisms causing intraocular infection. Endogenous endophthalmitis is less common, should be considered in the setting of fungemia, and is associated with specific risk factors [1]. Systemic immunosuppression without fungal bloodstream penetration is not considered a definitive risk factor; rather, the risk is in the combination of both. Examples of factors that can lead to penetration of the bloodstream by fungi include long-term indwelling intravenous catheters, prolonged hospital stays, abdominal surgery, and intravenous drug use. [4,5]. Figure 1 shows a case of endogenous fungal endophthalmitis, which was identified and correlated with a history of intravenous substance abuse, one of the definitive risk factors. Figure 2 also displays a case of endogenous fungal endophthalmitis, with a lesion characteristic of typical infection. When it comes to identifying the specific fungal species responsible for endogenous fungal endophthalmitis, the most frequently encountered organisms include *Candida albicans* and *Aspergillus* [6,7]. Other causative microorganisms include *C. dulineniesis*, *C. tropicalis*, and *A. niger* [6,7]. However, the precise proportions can vary depending on the data reported in the literature. In a study involving 27 cases of endogenous endophthalmitis, Binder et al. observed that among their 14 patients with fungal infections, 10 had *Candida albicans* infections (accounting for 71.4%), while 4 had A. fumigatus infections (28.6%) [8]. In contrast, Mir et al. examined a much larger sample of 56,839 cases of endogenous endophthalmitis and found that *Candida* infections were present in 6.7% of cases, while *Aspergillus* infections occurred in 0.4% of cases [5]. Additionally, in a study of 57 cases of endogenous endophthalmitis conducted by Lim et al., they identified *C. albicans* as the most common fungal cause, occurring in 9 cases (15.8%) [9]. Taken together, these findings suggest that yeasts, particularly *Candida* species, appear to be more commonly associated with endogenous fungal endophthalmitis, while molds, particularly *Aspergillus* and fusarium, are more frequently implicated in exogenous fungal endophthalmitis. However, it is important to note that a direct comparison or conclusive statement on this matter has not been formally studied or established.

Generally, there are two primary modalities of treatment for endophthalmitis. Firstly, medical management with the use of systemic with or without intravitreal antifungals is used to target the causative organism. Secondly, surgical management consists of removing the vitreous humor, known as pars plana vitrectomy. These management techniques have been discussed and reviewed in previous studies [2,3,4,5,6,7,8,9]. In this article, we aimed to review the role of intravitreal and systemic corticosteroids in fungal endophthalmitis as an adjuvant therapy to dampen the host inflammatory response to the fungal pathogen [1]. Intravitreal corticosteroids use is, however, controversial because of the possible detrimental effects of corticosteroids, such as their properties of impairment of the efficacy of antifungal drugs in vitro, as well as interference with the immunogenic response that potentially results in contrary fungal proliferation [10]. 

## 2. Methods

A systematic literature search was conducted to gather relevant studies on fungal endophthalmitis, corticosteroids, and related topics. The search was performed on two widely used academic databases, PubMed and Google Scholar. The search strategy involved using a combination of specific keywords related to the subject matter, including: “fungal endophthalmitis”, “corticosteroids”, “adjuvant”, “macrophage”, “immune response”, “yeast”, “phagocytosis”, “intravitreal”, and “dexamethasone”. The search was conducted up until September 2023, ensuring that the retrieved studies were recent and relevant to the current state of knowledge. Both titles and abstracts were screened during the initial phase to identify studies that appeared to be pertinent to the review’s objectives.

The inclusion criteria for studies comprised those published in the peer-reviewed English literature. The scope of inclusion encompassed various study types, including laboratory studies, clinical studies, and case reports. The decision to focus exclusively on English-language publications was intended to maintain consistency in language comprehension and ensuring a more standardized analysis of the selected studies. It is important to note that this systematic literature search aimed to identify studies that contributed valuable insights into the role of intravitreal and systemic corticosteroids in the context of fungal endophthalmitis. 

## 3. Pathobiology of Fungal Endophthalmitis Inflammation

The pathobiology of fungal endophthalmitis is thought to follow mechanisms similar to the innate immune defense of the retina and follow a similar progression of immune response of bacterial endophthalmitis and keratitis [11]. Research from Wayne State University by Gupta et al. showed this to be the case with an examination of *Aspergillus*-infected eyes in murine models. Using qRT-PCR, the researchers could assess toll-like receptor (TLR) expression following the induction of *Aspergillus fumigatus* endophthalmitis via intravitreal injection [12]. mRNA transcripts of TLR1, 2, 3, 4, 6, 7, 8, and 9 were found to be significantly elevated (*p* < 0.005) two days post-induction (DPI) [12]. Fungal ligands have been shown to interact with TLRs as a mechanism of innate immunity and induction of an inflammatory response [9]. Other than the expression of TLRs, the levels of inflammatory mediators were assessed by qRT-PCR and confirmed with ELISA, which showed that *Aspergillus*-infected retina displayed increased CXCL2, TNFα, IL-1β, and IL-6 [11]. The increased expression peaked at either 1 or 2 DPI, depending on the marker. This correlated with their data on fungal burden and neutrophil infiltration in infected eyes, which also peaked at 2 DPI before a gradual decline [12]. The pathway based on this research can be understood as an upregulation of TLR expression leading to the production of inflammatory mediators that recruit neutrophils to the site of infection, which then inhibit further growth of the pathogen [11]. Another investigation undertaken by this study dealt with neutropenic mice and assessed the difference in their response compared to controls. The results displayed the importance of neutrophils in the immune response against fungal (mold) endophthalmitis, as modified neutropenic mice had elevated fungal burden up to 5 DPI vs. 2 DPI in healthy controls and histologically corroborated increases in retinal tissue damage [12]. 

In a subsequent study, Rottmann et al. examined a murine model in a similar fashion, although inducing fungal (yeast) endophthalmitis using *Candida albicans* rather than *Aspergillus fumigatus* [13]. In this study, however, two different sets of mice, C57BL/6 and BALB/c mice, were compared to check for any strain-specific differences in immune response and fungal growth. Similar inflammatory markers were measured, including TNF-α, IL-1β, and IL-6 cytokines, as well as clinical scores grading levels of corneal haze/opacity and fungal burden [11]. Differences in pathology were observed, as BALB/c mice exhibited more intense pathology with higher clinical scores, a higher fungal burden, and more retinal damage and cell death than their C57BL/6 counterparts (*p* < 0.05) [13]. This was followed by further analysis, which showed that C57BL/6 had increased inflammatory cytokines and higher neutrophil infiltration (*p* < 0.05) and, therefore, a more robust innate immune response [13]. Finally, despite biological and pathologic differences between the two groups of mice, retinal function assessed via scotopic ERG showed no significant difference in retinal function between the two groups, which both had a severe decrease in visual function [13].

## 4. Anti-Inflammatory Action of Corticosteroids in Infection

While timely activation of the host immune system in infection and the occurrence of restricted inflammation is beneficial, persistent or excessive inflammation incites significant tissue damage. Corticosteroids, specifically glucocorticoids, have long been used in the history of medicine as inhibitors of inflammatory disorders, as well as being useful for their anti-inflammatory properties. This, combined with their immunosuppressive effect, has led them to be widely used therapeutic agents for many years. They are essential to treating many disease modalities, including adjuvant use in infections. While corticosteroids have pleiotropic mechanisms for combating inflammation, their mechanism of action relies on key molecular pathways mainly involving their direct effects on gene expression via binding glucocorticoid receptors [14]. Prostaglandin production is inhibited via the activation of annexin 1, which is upregulated and inhibits cytosolic phospholipase A_2_α, therefore blocking the release of arachidonic acid and the eventual production of prostaglandin [15]. Next, the glucocorticoid-to-glucocorticoid receptor interaction induces MAPK phosphatase I, which dephosphorylates and inactivates c-Jun and Fos, transcription factors that induce transcription of inflammatory and immune genes [16]. One additional pathway follows the cortisol–glucocorticoid receptor interaction’s blockade of NF-kB, which normally binds DNA sequences to stimulate the transcription of cytokines, chemokines, complement, and receptors for these molecules [17]. These mechanisms create a potent therapeutic agent but, unfortunately, may lead to clinically significant side effects with their use, necessitating judicial use.

## 5. In Vitro Effect of Corticosteroids on Immune Response against Fungal Infection

With the importance of innate immunity against fungal endophthalmitis, it is understandable that the adjuvant use of corticosteroids to dampen the severity of inflammation is controversial due to their possible interference with the host defense mechanisms against fungal infection. The limited literature studies investigating the in vitro effect have been compiled into a small timeline and included in Table 1, beginning with the mechanism of interference being described over several studies by Grasso et al. and Diamond et al., who showed that dexamethasone plays a role in the suppression of phagocytosis of yeasts and demonstrated the facilitation of yeast proliferation by dexamethasone [18,19,20,21,22].

Grasso et al. analyzed the inhibition of cell spreading and phagocytosis in cortisol-treated cultures of murine macrophages. A control group and a steroid-treated macrophage group were compared over a time frame of 6 days with regard to cell spreading and phagocytosis of heat-killed Saccharomyces cerevisiae [18]. The steroid-treated macrophages exposed to cortisol, methylprednisolone, dexamethasone, and triamcinolone acetonide were found to have slower rates of phagocytosis, reduced phagocytic indices (number of ingested yeast particles per macrophage), and cell spreading (measured based on the extension of cell processes) [18]. These results indicated corticosteroid inhibition of macrophage function for yeast phagocytosis and cell spreading.

Similarly, Grasso et al. investigated the ability of dexamethasone to suppress the ingestion of heat-killed Saccharomyces cerevisiae particles by stimulated murine macrophages. Stimulated macrophages represent cultured macrophages that have undergone differentiation, which is a variety of physiologic alterations, including increases in cell size, protein, and enzymatic activity [19]. Using measures of phagocytosis similar to those used in their 1981 study, Grasso et al. showed that the magnitude of the immune response in stimulated macrophages is increased when compared to cultures of resident macrophages [19]. They were then able to determine that the ingestion of yeast particles is inhibited in stimulated macrophages exposed to glucocorticoids, such as resident macrophages, leading to a conclusion that corticosteroids should, in theory, reduce host resistance in vivo [19]. 

After identifying the impact of corticosteroids on macrophage phagocytosis, Grasso and Becker sought to examine the mechanism of immune suppression. Over two studies, they examined that mechanism by creating a dialyzed medium from corticosteroid-treated macrophages and then treating macrophages from the dialysates of those cultures before analyzing phagocytic factors [20,21]. They identified a factor that they labeled phagocytosis inhibitory protein (PIP), which they hypothesized led to immune suppression [21]. The mechanism of action of this molecule was not precisely described. However, by analyzing cultures also treated with arachidonate, cyclooxygenase, and lipoxygenase inhibitors and describing no effect on the phagocytic capacity of macrophages, the researchers implied that the suppression of function was not associated with those pathways [20].

Another in vitro study performed by Diamond et al. studied the effects of steroid hormones on interactions of monocytes with hyphae. Three different concentrations of steroid hormones were examined, 10 µM, 1 µM, and 0.1 µM, with three different hyphal forms, *Candida albicans*, *Aspergillus fumigatus*, and *Rhizopus oryzae* [22]. Monocyte-mediated hyphal damage was inhibited significantly (*p* < 0.05) by all dosages of hydrocortisone with regards to *Candida* and *Aspergillus*, with no significant effect in rhizopus except at 10 µM [22]. It is understood that pharmacologic dosing of corticosteroids to achieve these concentrations is readily achievable in vivo. Therefore, pharmacologic doses of corticosteroids could interfere with the activity of host monocytes against the tissue-invasive forms of *C. albicans* and *A. fumigatus* [22].

The major limitation of the above-mentioned in vivo studies is the lack of treatment with antifungals and changes in vivo that are unaccounted for. Without antifungals being used simultaneously to test whether dexamethasone can provide benefit as an adjuvant therapy, no definitive conclusions can be drawn to rule out the possible benefit from faster clearance of inflammation or other possible advantages.

## 6. In Vivo Effect of Corticosteroids on Immune Response against Fungal Infection

There are only a limited number of studies that investigated the in vivo use of intravitreal steroids in animals or humans. The literature history of the in vivo effect of corticosteroids on immune response against fungal infection can be seen in Table 2. In 1974, Graham and Peyman investigated the intravitreal injection of dexamethasone for experimentally induced endophthalmitis [23]. They concluded that eyes treated with gentamicin and dexamethasone attained a significant reduction in inflammatory response leading to less severe damage of the anterior and posterior chambers, vitreous, retina, and choroid [23]. This was compared to eyes that were treated with gentamicin alone. However, their experiment was only carried out with regard to experimentally induced pseudomonas endophthalmitis. Still, it did serve as a baseline showing that the addition of corticosteroids could be an important adjunct to antimicrobial treatment [23].

Building on their previous work in 1974, Peyman and Coats analyzed fungal endophthalmitis. They utilized rabbit models to provide the initial evidence that adding corticosteroids did not impair antifungal activity or cause enhanced proliferation [21]. In the rabbit models with exogenous *Candida albicans* endophthalmitis, they found that in both treatment groups, only amphotericin B (*n* = 8) and amphotericin B plus dexamethasone (*n* = 8), had clearer vitreous after 4 days of treatment than controls (*n* = 4) [24]. At seven days, eyes treated with dexamethasone and amphotericin B displayed clearer vitreous as compared to eyes receiving only amphotericin B (*p* = 0.0017) [24].

In 1971, the prevailing idea surrounding the local administration of corticosteroids due to enhancing fungal growth was not only relevant to fungal endophthalmitis but also applied to other fungal eye infections, including fungal eye infections of the cornea. O’day et al. provided a case of a deep fungal corneal abscess treated with combined corticosteroid–antifungal therapy followed by penetrating keratoplasty that successfully resolved the fungal infection while preserving vision [25]. Their use of corticosteroids considered the risk of worsening intrinsic ocular disease. However, they decided during their specific case that it could not be made much worse and proceeded despite the possibility of systemic spread, which they deemed improbable in an immune-competent patient [25]. The high doses of corticosteroid they utilized were tolerated, and a good visual result was achieved, leading to this study being cited by future studies as a basis for steroid use in similar fungal eye infections [25]. They also predicted that with the advent of more effective antifungal agents, corticosteroid use would become more prevalent in fungal eye infections and may be used earlier in treatment.

The effect of this study can be seen in studies such as the case reports by Elliot et al. in 1979, describing two cases of fungal endophthalmitis in drug abusers who were treated not only with appropriate antifungals but also with corticosteroids [26]. They cited the results of O’day et al. as justification for their judicious use, as they believed them to be mandatory in certain conditions, especially to achieve and preserve clear visual function. However, this embodies one of a few reports of a similar kind, which all cite the lack of management guidelines and rules regarding appropriate timing for corticosteroids, their length of treatment, and even the route to be used. In the end, however, they concluded that corticosteroid use and timing are crucial in some specific clinical situations, such as fungal endophthalmitis or fungal corneal disease, especially during the management of complex cases [26].

In a retrospective study by Majji et al., the role of adjunctive dexamethasone in fungal endophthalmitis was examined [27]. This represented one of the first larger studies as twenty cases of culture-proven exogenous fungal endophthalmitis were analyzed for differences in visual and anatomic outcomes. There were no statistically significant differences in either group [27]. The rate of inflammation clearance was also measured, with the adjuvant steroid group showing faster clearance, although it was irrespective of fungal growth [27]. Thus, other factors need to be analyzed, ideally with a prospective study, to determine the possible benefit of steroids. Identification of causative agent with appropriate sensitivity to antifungals, and the institution of these treatments, need to be established before using steroids [27]. Afterward, the dosage and timing of steroids are still important factors to consider.

In another series of 27 cases of culture-proven fungal endophthalmitis treated with intravitreal amphotericin B and dexamethasone with additional interventions in some cases [28]. They recognized corticosteroid use as controversial. However, their cases achieved functional success in 37.04% of cases, with the contemporary literature reporting anywhere between a 0–44% rate of successful functional outcomes [28]. They maintained similar reasoning behind their corticosteroid use to previously mentioned studies, namely to control tissue destruction secondary to host defense mechanisms, including neutrophils. They theorized that the size of fungal hyphae was responsible for preventing ingestion by neutrophils but would not inhibit the release of lysosomal enzymes or damaging oxygen metabolites, which could be prevented by steroid treatment [28]. Regarding the possible damaging effects of corticosteroids, it was stated that the danger of steroids generally lies in the usage of high corticosteroid dosing and use as monotherapy without proper antifungal medication coverage. However, corticosteroid use as adjunctive treatment to antifungal agents is appropriate.

A more recent study by Corredores et al. analyzed the effect of different treatment modalities on visual outcomes in patients with fungal endophthalmitis, with one of the factors evaluated being the use of oral/intravitreal steroids [29]. The general trend observed was that eyes treated with steroids benefited from greater visual acuity, with a logMAR change from 1.2 (Snellen equivalent. 20/320) at the initial visit to 0.6 (Snellen equivalent. 20/80), compared to eyes that did not receive dexamethasone, which had a logMAR VA change from 0.7 (Snellen equivalent. 20/100) to 0.4 (Snellen equivalent. 20/50) [29]. While the difference was not statistically significant, these results corroborate the findings of previous studies by Majji et al. and Coats and Peyman. However, the number of patients treated with steroids was only five in total, with all receiving oral corticosteroids. At the same time, two received intravitreal dexamethasone, and the total number of patients analyzed in the study was thirteen [29]. A conclusion was drawn that the benefit of corticosteroids cannot be ruled out. While their use may not be standard of care currently, they should be considered for patients with a prolonged disease course or those with severe inflammation to help limit inflammation-related damage [29]. 

In one of the few studies with statistics mentioned on the subject, Sallam et al. showed that systemic corticosteroids could potentially increase the risk of severe visual loss associated with fungal endophthalmitis, though they did not report a statistically significant result and with possible confounders [30]. Similar non-statistically significant results were reported by Sen et al. in 2021 when analyzing post-surgical cases of fungal endophthalmitis, but an opposite trend was also observed, namely of better vision in eyes receiving intravitreal steroids (*n* = 9) having a final logMAR BCVA of 1.32 (Snellen equivalent. 20/400) and eyes not receiving intravitreal steroids (*n* = 8) having a final logMAR BCVA of 2.21 (Snellen equivalent. 20/800), *p* > 0.05 [31]. However, current practice is that systemic corticosteroids should be avoided in fungal endophthalmitis, a sentiment that has been echoed recently, with further studies necessary for any recommendation changes [30]. Of note, the differences in the pharmacokinetics of systemic steroids vs. intravitreal steroids must be considered because the ocular bioavailability of intravitreal steroids is much higher than systemic steroids. One other factor that should be considered is the possible masking of signs and symptoms of infection, which have been reported in fungal endophthalmitis previously, as this may delay diagnosis and treatment and worse outcomes overall [32]. In this context, the effect of intravitreal steroids is difficult to reverse compared to systemic steroids that can be stopped.

**Table 2:** Timeline of in vivo studies regarding effects of corticosteroid effect on fungal infection.

In Vivo Timeline	1971: O’Day and colleagues provided a case of a deep fungal corneal abscess treated with combined steroids and antifungal that resolved the infection while preserving vision [26].	1974: Graham and Peyman investigated utilization of intravitreal injection of dexamethasone for experimentally induced endophthalmitis and concluded that eyes treated with gentamicin and dexamethasone attained a significant reduction in inflammatory response [23].	1979: Elliott JH, and O’Day published two cases of fungal endophthalmitis in drug abusers who were treated with appropriate antifungals but also corticosteroids [27].
1992: Coats and Peyman utilized rabbit models with exogenous *Candida albicans* endophthalmitis and found at seven days, eyes treated with dexamethasone and amphotericin B displayed clearer vitreous as compared to eyes receiving only amphotericin B [24].	1999: Majji at al. analyzed the role of adjunctive dexamethasone in fungal endophthalmitis, as twenty cases of culture-proven exogenous fungal endophthalmitis were analyzed for differences in visual and anatomic outcomes, but no statistically significant differences were found with/without adjuvant steroids [27].	2001: Narang and colleagues described 27 cases of culture-proven fungal endophthalmitis treated with intravitreal amphotericin B with additional dexamethasone interventions in some cases, these cases achieved functional success in 37.04% of cases, with the contemporary literature reporting anywhere between a 0–44% [28].	2021: Sen et al. analyzied post-surgical cases of fungal endophthalmitis and noted a trend of better vision in eyes receiving intravitreal steroids but results were non-statistically significant [31].

## 7. Discussion

Treatment of fungal endophthalmitis has been well studied, including the role of systemic and intravitreal antifungal agents and pars plana vitrectomy surgery [1,2,3,4,5,6,7,8,9]. The use of corticosteroids as an adjunctive treatment is supported by in vitro studies to decrease tissue damage resulting from an upregulated immune system due to their anti-inflammation properties [18,19]. However, there remains at least a theoretical concern from ophthalmologists that the use of corticosteroids results in a decrease in the efficacy of antifungal drugs or interference with the host immunogenic response and microbiologic recovery that potentially results in fungal proliferation and disease worsening [31,32]. 

In this review, we found that only a small number of studies have retrospectively investigated fungal endophthalmitis management with the use of adjuvant steroids in oral or intravitreal forms in addition to the antifungal treatment and/ or vitrectomy surgery [29,30]. The outcomes of the patients in these studies were mixed and unstratified, thus difficult to discern any definitive results. However, these analyses [33,34] still echo the notion by Coats and Peyman [24] as well as Majji et al. [27] regarding the possible benefit of steroids for inflammatory clearance and the concerns of making signs of infection deterioration due to interference with the host immune responses [31,32].

With regards to systemic corticosteroids, there are two separate entities of discussion required, their possible role in acting as a risk factor for endogenous endophthalmitis, as well as the potential benefits that can be extrapolated from the effect of intravitreal corticosteroids, which showed a positive effect on inflammatory clearance in fungal endophthalmitis. There exists a distinct paucity of studies that primarily investigate either of these notions. However, they have both been anecdotally noted in larger analyses [35,36,37]. It has been mentioned previously that while the use of corticosteroids remains controversial in all forms of endophthalmitis, specifically fungal endophthalmitis represents an area of study with very little evidence to help guide treatment [38].

A main limiting factor of many studies that have currently associated systemic steroid use with the development of fungal endophthalmitis is the inability to separate potential confounding factors, such as the underlying condition for which the systemic steroids were started. One such example exists with a retrospective investigation of endogenous fungal endophthalmitis following systemic corticosteroid administration to treat COVID-19 [39]. Patients in this study were explicitly hospitalized within the ICU for COVID-19-associated illness for an average of 18.2 days and had received systemic steroids for an average of 42 days [39]. While they reported prolonged steroid therapy as a probable contributing factor to infection, they also recognized that COVID-19 infection could lead to an immunocompromised-like state, and many risk factors are associated with prolonged hospital stays, particularly in the ICU, such as indwelling intravenous lines that may have acted as probable sources of infection [39]. A recent study reviewing endogenous fungal endophthalmitis defined the risk factors as systemic conditions, which included immunosuppressive therapy as one of the possible predisposing factors, but also listed diabetes mellitus, recent hospitalization, indwelling lines, respiratory disease, liver disease, renal disease, malignancy, and more as contributing factors [40]. Considering the prevalence of these risk factors, controlling for all of these would be challenging, and thus hard to isolate the true impact of corticosteroids on disease risk. A prospective randomized control study is warranted.

While there have been only a limited number of studies examining steroids as adjuvant treatment in fungal endophthalmitis, there have been a few high-quality studies regarding steroids in a similar use during bacterial endophthalmitis. This is important in evaluating possible benefits to be drawn from similar studies for fungal endophthalmitis and represents necessary steps to drawing conclusions and establishing the mainstays of treatment. In 2022, a review of four randomized controlled trials comparing the effectiveness of adjunctive steroids versus antibiotics alone in managing acute bacterial endophthalmitis suggested a possible improvement in visual outcomes at 3 months when using adjunctive steroids, although this result was not statistically significant [41]. Their conclusion also contained suggestions for future studies which might be applied broadly and utilized in any future studies which might investigate adjuvant steroids in fungal endophthalmitis. These suggestions included examining specific clinical settings, focusing and stratifying based on causative organism or etiology, and also including outcomes based on patient symptomology and establishing uniform outcomes that are consistent between studies short- and long-term intervals [41].

In conclusion, given the limited literature present and the heterogeneity of the existing studies, additional clinical trials or observations in humans over long periods need to be done analyzing the effect of systemic and intravitreal corticosteroids in fungal endophthalmitis before definitive conclusions can be drawn regarding their risk/ benefit as an adjuvant to antifungal agents. 

## Figures and Tables

**Table 1:** Timeline of in vitro studies regarding effects of corticosteroid on fungal infection.

In Vitro Timeline	1981: Grasso et al.’s results indicated corticosteroid inhibition of macrophage function for yeast phagocytosis and cell spreading [18].	1982: Grasso et al. determine that the ingestion of yeast particles is inhibited in stimulated macrophages exposed to glucocorticoids [19].
1983: Diamond showed corticosteroids could interfere with the activity of host monocytes against the tissue-invasive forms of *C. albicans* and *A. fumigatus* [22].	1985: Becker and Grasso demonstrated inhibition of phagocytosis and cell spreading may be mediated by a dexamethasone-induced non-dialyzable factor [20].	1988: Becker and Grasso suggested that the suppression of yeast phagocytosis by dexamethasone action may be associated with the inhibition of phospholipase A2 activity [21].

## Data Availability

Not applicable.
